# Assessment of 3-Dimensional vs 2-Dimensional Imaging and Technical Performance Using a Multiport Intraoperative Data Capture and Analytic System for Patients Undergoing Laparoscopic Roux-en-Y Gastric Bypass Surgery

**DOI:** 10.1001/jamanetworkopen.2019.20084

**Published:** 2020-01-29

**Authors:** Mauricio E. Gabrielli, Tomas J. Saun, James J. Jung, Teodor P. Grantcharov

**Affiliations:** 1 International Centre for Surgical Safety, St Michael’s Hospital, Toronto, Ontario, Canada; 2 Keenan Centre for Biomedical Research, St Michael’s Hospital, Toronto, Ontario, Canada; 3 Department of Surgery, University of Toronto, Toronto, Ontario, Canada; 4 Division of Plastic and Reconstructive Surgery, Department of Surgery, University of Toronto, Toronto, Ontario, Canada; 5 General Surgery, St Michael’s Hospital, Toronto, Ontario, Canada

## Abstract

**Importance:**

Errors and adverse events occur frequently in health care. Three-dimensional (3-D) laparoscopic systems claim to provide more realistic depth perception and better spatial orientation compared with their 2-D counterparts.

**Objective:**

To compare the association of 3-D vs 2-D systems with technical performance during laparoscopic Roux-en-Y gastric bypass (LRYGB) procedures using a multiport intraoperative data capture system.

**Design, Setting, and Participants:**

This cohort study was performed between May and December 2018, with a total of 50 LRYGB procedures performed in an academic tertiary care center; recordings of the operations were evaluated with a 30-day follow-up. All procedures were performed by the same surgical team.

**Exposure:**

Surgical teams used 2-D or 3-D laparoscopic systems.

**Main Outcomes and Measures:**

Technical performance was evaluated using the Objective Structured Assessment of Technical Skill and surgical errors and events using the Generic Error Rating Tool.

**Results:**

Of the 50 patients who underwent LRYGB procedures, 42 (86%) were women, with a median (interquartile range) age of 42 (35-47) years and a median (interquartile range) body mass index of 46 (42-48), with no significant demographic differences between the groups whose operations were performed using the 2-D and 3-D systems. The mean (SD) number of errors per case was significantly lower in procedures using the 3-D laparoscopic system than in those using the 2-D system (17 [6] vs 33 [2]; *P* < .001). The mean (SD) number of error-related events was significantly lower in procedures using the 3-D system than in those using the 2-D system (6 [2] vs 11 [4]; *P* < .001). Mean (SD) Objective Structured Assessment of Technical Skill scores were significantly higher when the 3-D system was used than when the 2-D system was used (28 [4] vs 22 [3]; *P* < .001).

**Conclusions and Relevance:**

In this limited sample of LRYGB procedures, the use of a 3-D laparoscopic system was associated with a statistically significant reduction in errors and events as well as higher Objective Structured Assessment of Technical Skill scores compared with 2-D systems.

## Key Points

**Question:**

Is the adoption of a 3-dimensional (3-D) vs 2-D laparoscopic system associated with significantly different levels of technical performance during laparoscopic Roux-en-Y gastric bypass procedures?

**Findings:**

In this cohort study of 50 Roux-en-Y procedures, there were significantly fewer technical errors, significantly fewer error-related events, and significantly higher Objective Structured Assessment of Technical Skills scores when the 3-D system was used.

**Meaning:**

In this study, a 3-D laparoscopic system was associated with a higher level of technical performance among surgeons performing a Roux-en-Y procedure.

## Teaser

This cohort study compares technical performance associated with the use of 3-dimensional (3-D) vs 2-D imaging during laparoscopic Roux-en-Y gastric bypass procedures using a multiport intraoperative data capture system.

## Introduction

Multiple reports have indicated that errors and adverse events happen frequently in modern health care and result in significant morbidity and mortality as well as increased health care costs.^1,2,3,4^ Large investments have been made in technology and data collection; however, these have not contributed to statistically significant reductions in adverse events.^5^

When evaluating closed malpractice cases, technical errors are the most frequently described type of surgical error, and these have been analyzed in several retrospective reviews.^6,7^ Our research group^8^ has shown that the use of the Generic Error Reporting Tool (GERT) in conjunction with a synchronized multiport data recording system provides an objective, reliable, and easy-to-use tool for assessment of surgical errors and surgical performance. Previous work has shown that technical performance assessed by GERT predicts outcomes in laparoscopic surgery.^9^

Not all technical errors lead to intraoperative events. The identification and root cause analysis of technical errors is critical in preventing the occurrence of intraoperative events and mitigating the likelihood of postoperative complications and clinically significant adverse outcomes.

Others studies^10,11,12^ have shown that between 39.6% and 54.2% of medical adverse events occur in the operating room (OR). In laparoscopic cases, a substantial number of these events are associated with depth perception, as evidenced by a study that evaluated 252 cases of laparoscopic cholecystectomy, in which 97% of surgical accidents occurred as a result of visual misperceptions.^13,14,15,16^ In the past 20 years, the advancement of laparoscopic technology has led to increased safety, better precision, and improved efficiency in the OR, but the lack of depth perception when using traditional 2-dimensional (2-D) laparoscopic systems remains a major limitation.^17^

Three-dimensional laparoscopic systems provide direct stereoscopic depth, which may improve operative speed and technical performance in laparoscopic procedures. Numerous studies^18,19^ have shown that 3-D laparoscopic systems can decrease intraoperative blood loss, operative time, and the surgeon’s learning curve; however, most of these studies used a nonliving system. Despite this potential for improved surgical safety, the use of 3-D laparoscopic equipment remains limited in modern surgical centers.^13,17,20^ This could be explained by the high cost of this technology and the limited evidence of the utility of 3-D systems in minimally invasive surgery.^21^ A previous study^22^ has explained the underlying scientific reasons for the apparent improvement in 3-D surgical task performance. However, this improvement comes with increased eye strain, headache, and other visual symptoms.^23,24^ The literature^25,26,27,28^ has evaluated the consequences of 3-D imaging systems in laparoscopic surgery, but prospective data are limited, lacking robust objective evaluation criteria for technical performance or evaluation of clinically significant patient outcome measures. The purpose of this study was to compare the association of using 3-D vs 2-D imaging systems with technical performance and surgical safety while performing laparoscopic Roux-en-Y gastric bypass (LRYGB) procedures.

## Methods

This study was approved by the research ethics board at St. Michael’s Hospital, and all study participants provided explicit written consent. The institutional review board approved 2 of us (T.P.G. and M.E.G.) participating in the study, given that we were not involved in the analysis of the procedures. This study followed the Strengthening the Reporting of Observational Studies in Epidemiology (STROBE) reporting guideline.

A total of 50 consecutive LRYGB procedures were performed by the same surgical team (ie, same surgeon [T.P.G.] and same fellow [M.E.G.] for all procedures) in an OR equipped with the Operating Room Black Box (Surgical Safety Technologies), a synchronized multiport data recording system that prospectively captures and records surgical procedures from multiple data sources, allowing for objective assessment of the OR environment. The synchronized multiport data recording system collects data from the OR, including video and audio recordings, patient physiology, environmental factors, and devices used. These data are processed by expert surgical analysts, using instruments such as the Objective Structured Assessment of Technical Skill (OSATS) and the GERT to output metrics including technical performance and intraoperative errors and events. The clinical applications of this technology are numerous and diverse; they include improved surgical training, quality, and patient safety using high-quality intraoperative data.^29^

The study was conducted at an academic tertiary care facility. The first 25 procedures used a high-definition 2-D laparoscopic video system (Olympus), and the subsequent 25 procedures used a high-definition 3-D laparoscopic video system (Olympus). During the trial, all jejunojejunostomies were performed by the fellow (M.E.G.), and the gastric pouches and gastrojejunostomies were performed by the staff surgeon (T.P.G.).

The surgical team had performed more than 500 laparoscopic procedures using a 2-D system and more than 100 procedures using a 3-D laparoscopic system before the trial; however, a 1-hour standardized training session on using the 3-D laparoscopic system was provided to all participants, and 3 LRYGB procedures were performed with the 3-D laparoscopic video system before study commencement.

Inclusion criteria for patients included being older than 18 years, undergoing an elective LRYGB procedure, and having a body mass index (BMI, calculated as weight in kilograms divided by height in meters squared) between 35.0 and 54.9. Exclusion criteria included prior open abdominal surgery, prior laparoscopic upper gastrointestinal surgery, and inability or unwillingness to provide informed consent.

The surgical team included a staff surgeon (T.P.G.) and first assistant (M.E.G.), and informed consent was obtained from these individuals for the duration of the study. Because the synchronized multiport data recording system collects data on all individuals in the operating room, informed consent was also obtained from nursing staff, the anesthesia team, and any trainees present. To foster perioperative team engagement, approval was obtained from the clinical manager of perioperative services (ie, the OR leader), and anesthesia staff were invited to join an informative presentation about the study.

### Data Collection

All 50 LRYGB procedures were performed between May and December 2018. For all eligible procedures, audio and video data were continuously recorded, with a follow-up of 30 days for all cases. Wall-mounted cameras captured a panoramic view of the entire OR, and the laparoscopic camera captured the intracorporeal view. Data capture commenced after the patient was intubated and ended just before extubation. During the entirety of data capture, the patient was draped so that only the operative field was visible. All data feeds were stored on a secure server. Patient information, including demographic data, relevant diagnoses, and risk factors, were collected from the electronic health record.

### Evaluation of Recorded Procedures

#### Surgical Technical Skills

Technical skills were evaluated by expert surgical analysts who were not involved with the surgical team and who were masked to the visualization system used during the surgery, given that their review used standard 2-D monitors using the OSATS tool.^30^ In addition, the GERT^8^ was used to identify intraoperative errors and events. All expert surgical analysts underwent formal training in surgical case analysis, and the interrater reliability of their measurements was periodically assessed.

Technical errors were defined as failure to complete a planned action as intended or the use of an incorrect plan to achieve an aim. Technical errors may occur in the absence of adverse events.^31^ The GERT classifies technical errors into 4 error modes: (1) too much use of force or distance, (2) too little use of force or distance, (3) inadequate visualization, and (4) incorrect orientation of instrument or dissection plane. Each error mode could be encountered in a variety of different surgical tasks, including abdominal access, use of retractors, use of energy devices, grasping and dissection, cutting, transection and stapling, clipping, suturing, and use of suction. This type of error classification has been used in multiple studies investigating technical errors within a hierarchical task analysis framework. Events were defined as any deviation from the standard intraoperative course that caused injury (such as thermal or mechanical tissue injury).^8,32^ Depth perception errors occurred when the root cause of the error was associated with distance, which would include the first 2 GERT error modes. Depth perception was a specific metric and was measured for this study using a survey. It was not associated with the data captured by the synchronized multiport data recording system. Low technical performance was defined as an OSATS score less than 22. The threshold values for technical performance were chosen based on previous studies.^8,33,34^

#### Clinically Significant Adverse Outcomes 

Clinically significant adverse outcomes were defined as any deviation from the standard surgical or postoperative course that required special treatment or rectifying actions and were classified as either related or not related to the 3-D laparoscopic image system (ie, root of the event related or not related to image quality). All clinically significant adverse outcomes were assessed using the Clavien-Dindo classification.^35^

### Statistical Analysis

All statistical analyses were performed using SPSS statistical software version 22 (IBM Corp). Normality of data distribution was assessed using the Shapiro-Wilk test. Descriptive statistics were performed. Continuous variables were compared using the *t *test or Mann-Whitney *U* test, and categorical variables were compared using the χ^2^ test of association. Significant variables from the univariate analysis were included in a multivariable logistic regression analysis controlling for age, sex, BMI, and comorbidities (Charlson Comorbidity Index). Statistical significance was set at *P* < .01, and all tests were 2-tailed.

## Results

Of 50 patients enrolled, 42 (86%) were women, with a median (interquartile range [IQR]) age of 42 (35-47) years and a median (IQR) BMI of 46 (42-48). Patient demographic characteristics were comparable between groups (Table 1).

**Table 1.** Patient Demographic Characteristics

Characteristic	No. (%)			*P* Value
	All Cases (N = 50)	2-D System (n = 25)	3-D System (n = 25)	
Age, median (IQR), y	42 (35-47)	41 (33-46)	43 (37-53)	.31
Women	42 (86)	23 (92)	19 (76)	.25
BMI, median (IQR)	46 (42-48)	46 (42-48)	44 (40-49)	.56
CCI score				
0	17 (34)	9 (36)	8 (32)	.66
1	16 (32)	9 (36)	7 (28)	
2	12 (24)	4 (16)	8 (32)	
3	5 (10)	3 (12)	2 (8)	

Abbreviations: 2-D, 2-dimensional; 3-D, 3-dimensional; BMI, body mass index (calculated as weight in kilograms divided by height in meters squared); CCI, Charlson Comorbidity Index; IQR, interquartile range.

The total number of technical errors and the mean error rate were both significantly lower when the 3-D laparoscopic system was used compared with when the 2-D laparoscopic system was used. When the 3-D system was used, there were 416 total errors, with a mean (SD) of 17 (6) errors per case; when the 2-D system was used, there were 834 total errors, with a mean (SD) of 33 (2) errors per case (*P* < .001).

**Figure 1.**
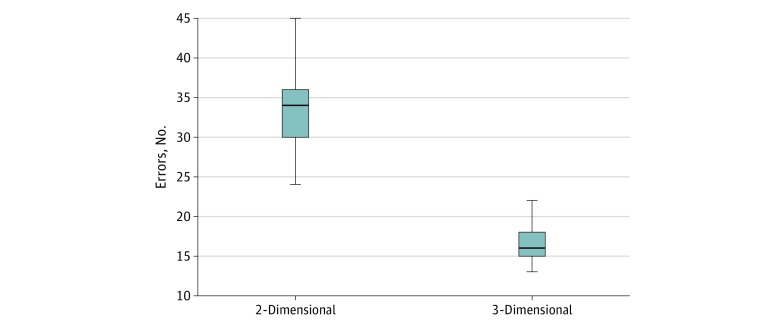
Total Number of Technical Errors per Case The middle lines in the boxes indicate the medians, with the upper and lower bounds representing the interquartile range. Whiskers indicate the minimum and maximum values.

**Figure 2.**
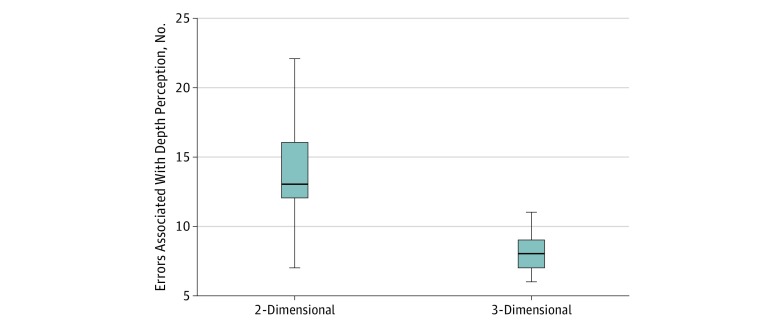
Total Number of Technical Errors Associated With Depth Perception per Case The middle lines in the boxes indicate the medians, with the upper and lower bounds representing the interquartile range. Whiskers indicate the minimum and maximum values.

The total number of technical errors per case as well as errors per case associated with depth perception were significantly lower when the 3-D laparoscopic system was used compared with when the 2-D system was used. When the 3-D system was used, there was a mean (SD) of 17 (6) errors per case, with a mean (SD) of 8 (1) errors associated with depth perception. When the 2-D system was used, there was a mean (SD) of 33 (2) errors per case, with a mean (SD) of 14 (3) errors associated with depth perception (*P* < .001) (Figure 1 and Figure 2).

The number of technical errors per case when suturing, both in total and associated with depth perception, were significantly lower when the 3-D system was used compared with when the 2-D system was used. When the 3-D system was used, there was a mean (SD) of 11 (2) errors per case, with a mean (SD) of 7 (1) errors associated with depth perception. When the 2-D system was used, there was a mean (SD) of 19 (4) total errors per case, with a mean (SD) of 11 (1) errors associated with depth perception (*P* < .001).

**Figure 3.**
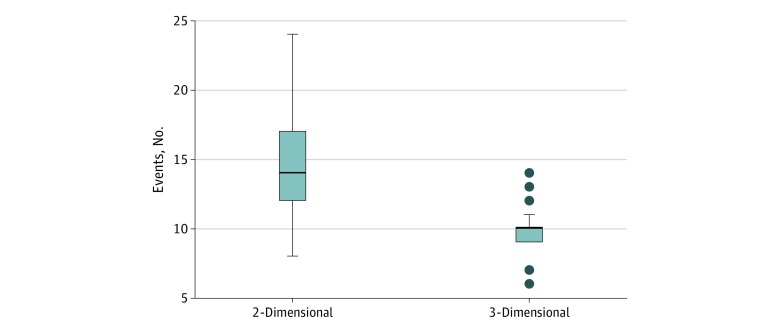
Total Number of Adverse Events per Case The middle lines in the boxes indicate the medians, with the upper and lower bounds representing the interquartile range. Whiskers indicate the minimum and maximum values, and dots indicate outliers.

The mean (SD) number of events per case was significantly lower with the 3-D system than with the 2-D system (10 [2] vs 15 [4] events/case; *P* < .001) (Figure 3). There was a significantly lower mean (SD) number of error-related events in procedures using the 3-D laparoscopic system compared with procedures using the 2-D system (6 [2] vs 11 [4]; *P* < .001); however, there was no difference in the number of events not related to errors (*P* = .87) (eFigure in the Supplement).

The OSATS scores were significantly higher when the 3-D system was used compared with when the 2-D system was used. The mean (SD) OSATS score with the 3-D system was 28 (4) vs 22 (3) with the 2-D system (*P* < .001).

The median (IQR) operative duration for all cases was 85 (75-95) minutes. When the 3-D system was used, the median (IQR) operative duration was significantly lower than when the 2-D system was used (80 [70-90] minutes vs 93 [85-100] minutes; *P* < .001).

Controlling for age, sex, BMI, and comorbidities, multivariable regression models were constructed to evaluate whether using the 3-D laparoscopic system was associated with the number of technical errors, number of events, operative duration, and OSATS scores. The use of the 3-D system was the only statistically significant independent factor associated with a lower number of technical errors (estimate, −17 errors; *P* < .001), a lower number of events (estimate, −5.14 events; *P* < .001), and a higher OSATS score (estimate, 5.9 points; *P* < .001) (Table 2).

**Table 2.** Multivariable Regression Model

Outcome	Estimates	*P* Value
Errors, No.		
3-D vs 2-D	−17	<.001
Age	0.038	.69
Women	1.62	.40
BMI	−0.08	.95
CCI 1 vs 0	−2.26	.16
CCI 2 vs 0	0.86	.64
CCI 3 vs 0	−2.42	.37
Events, No.		
3-D vs 2-D	−5.137	<.001
Age	−0.017	.27
Women	−1.16	.06
BMI	−0.067	.05
CCI 1 vs 0	−0.21	.04
CCI 2 vs 0	0.28	.02
CCI 3 vs 0	1.34	.05
OSATS Score		
3-D vs 2-D	5.9	<.001
Age	0.035	.76
Women	−1.71	.08
BMI	0.042	.05
CCI 1 vs 0	0.95	.05
CCI 2 vs 0	−1.84	.05
CCI 3 vs 0	0.27	.38
Procedure Duration, min		
3-D vs 2-D	−13.4	.02
Age	−0.3	.40
Women	−3.51	.05
BMI	−0.87	.03
CCI 1 vs 0	−10.86	.03
CCI 2 vs 0	−9.09	.05
CCI 3 vs 0	−7.85	.52

Abbreviations: 2-D, 2-dimensional; 3-D, 3-dimensional; BMI, body mass index, CCI, Charlson Comorbidity Index; OSATS, Objective Structured Assessment of Technical Skill.

Only 1 clinically significant adverse event was identified during the course of this study. One patient, for whom the 3-D system was used, developed a Clavien-Dindo grade IIIb complication because of bleeding from the staple line, which is considered an event not related to a technical error. Therefore, during this study, there were no clinically significant adverse events associated with the use of the 3-D laparoscopic system.

## Discussion

This study objectively demonstrated that the use of a 3-D laparoscopic system was associated with an improvement in technical performance among surgeons performing LRYGB procedures. When the 3-D laparoscopic system was used, the number of errors per case was significantly lower, there were significantly fewer error-related events, and the OSATS scores were significantly higher. After controlling for age, sex, BMI, and comorbidities, the use of a 3-D laparoscopic system remained an independent variable associated with better technical performance.

Three-dimensional laparoscopic systems have been around since the 1990s; however, the enthusiasm to use this technology quickly subsided because they caused adverse effects in surgeons, such as eye strain and headache.^23^ Modern high-definition 3-D laparoscopic systems also present challenges: there are no 5 mm scopes, specific glasses need to be worn, and the monitor needs to be optimally positioned to avoid disturbances.^16^

Other studies^28,36,37^ have shown technical improvement using 3-D laparoscopic systems, but the results have been inconsistent. A prospective, randomized clinical trial^16^ that compared a 3-D system with a 2-D system for laparoscopic cholecystectomy did not show any improved technical performance when the 3-D system was used. However, another study^36^ concluded that the 3-D system provided advantages during the execution of technical tasks that demand a high degree of spatial perception, such as suturing and knot tying. The LRYGB procedure requires advanced laparoscopic technical skills and high spatial perception during surgery; this could explain why the results were robustly in favor of the 3-D laparoscopic system in our study.

In this study, the number of technical errors per case associated with depth perception represented more than 40% of the technical errors per case in both groups. Therefore, the proportionate reduction in technical errors per case associated with depth perception when the 3-D system was used suggests that the benefits of the 3-D system extended beyond depth perception–associated errors. This suggests that the use of a 3-D laparoscopic system may have value beyond improving spatial perception in the laparoscopic surgical field.

The total number of error-related events was significantly lower when the 3-D system was used; however, there was no difference in the number of events not related to technical errors. This suggests that the 3-D system may improve surgical safety by reducing the proportion of technical errors that cause events. A previous study^38^ that compared technical performance in laparoscopic gastric cancer surgery using 3-D vs 2-D imaging systems showed that there was more intraoperative bleeding when the 2-D system was used; however, the cause of the intraoperative bleeding was never identified. The present study was able to attribute specific intraoperative events to specific technical errors, creating a unique opportunity to develop future strategies for mitigation.^36^

In this study, the OSATS scores in both groups were in the range of high technical performance based on previous research.^8,33,34^ This is an interesting finding because it contradicts the finding of significantly different technical performance as evaluated by the GERT,^8,30^ and the evaluation of all metrics was performed by the same group of expert surgical analysts. This may be because the OSATS is more subjective and less granular than the GERT. Furthermore, because the OSATS is scored after the entire procedure and procedure steps are reviewed, there exists a recall bias compared with the GERT, where errors and events are scored as they are objectively observed. Based on these findings, it is important to consider a multimodal assessment of technical performance, given that single instruments can be hard to interpret alone.

We found an association with shorter operative duration when the 3-D system was used. Previous studies comparing 3-D and 2-D systems^23,25,26,27,39,40^ have reported mixed results regarding operative duration. This lack of consistency may be explained by the diversity of 3-D systems used in the different studies^17^ and possibly by differences in the technical skills among surgeons.

### Limitations

This study has limitations. It took place in a single institution, with a single surgical team, and only assessed a single surgical procedure. As different laparoscopic procedures may have variable demands for depth perception, the results of this study may not be generalizable across different procedure types. Furthermore, this study only looked at a single 3-D system. There are other types of 3-D laparoscopic systems in use, and these results may not be generalizable across all systems.^27,40,41^

As is common with research involving the adoption of new technology, a series of consecutive cases were reviewed in this study.^42^ It is understood that this convenience sampling brings with it inherent bias because the results from the cohort after the new technology was introduced were performed by a more experienced surgical team. However, the surgeons in this study already had a significant amount of experience with LRYGB procedures; therefore, this effect is expected to be minimal. However, some data suggest that the 3-D visualization system is more favorable at the beginning of the learning curve.^20^ Further research may benefit from conducting multicenter, multisurgeon trials with different types of laparoscopic procedures. This would provide more robust evidence for the potential benefits of this technology in terms performance, safety, and cost.

## Conclusions

To our knowledge, this study was the first to compare a 3-D laparoscopic system with a 2-D system using an objective intraoperative data capture platform. In this limited sample, 3-D technology demonstrated a statistically significant reduction in errors and events and an improvement in OSATS scores in LRYGB procedures compared with 2-D systems.

## Notes

Supplement. **eFigure. **Number of Events Not Related to Error
