# Supplementary material for: Assessment of 3-Dimensional vs 2-Dimensional Imaging and Technical Performance Using a Multiport Intraoperative Data Capture and Analytic System for Patients Undergoing Laparoscopic Roux-en-Y Gastric Bypass Surgery
Source: JAMA Netw Open. 2020 Jan 29;3(1):e1920084. doi: 10.1001/jamanetworkopen.2019.20084 (PMC6991293; doi:10.1001/jamanetworkopen.2019.20084)
Supplement: Supplement. — eFigure. Number of Events Not Related to Error [file jamanetwopen-3-e1920084-s001.pdf]

Supplementary Online Content

Gabrielli ME, Saun TJ, Jung JJ, Grantcharov TP. Assessment of 3-dimensional vs 2-dimensional imaging and technical performance using a multiport intraoperative data capture and analytic system for patients undergoing laparoscopic Roux-en-Y gastric bypass surgery. *JAMA Netw Open*. 2020;3(1):e1920084. doi:10.1001/jamanetworkopen.2019.20084

eFigure. Number of Events Not Related to Error

This supplementary material has been provided by the authors to give readers additional information about their work.

© 2020 Gabrielli ME et al. *JAMA Network Open.*

eFigure. Number of Events Not Related to Error

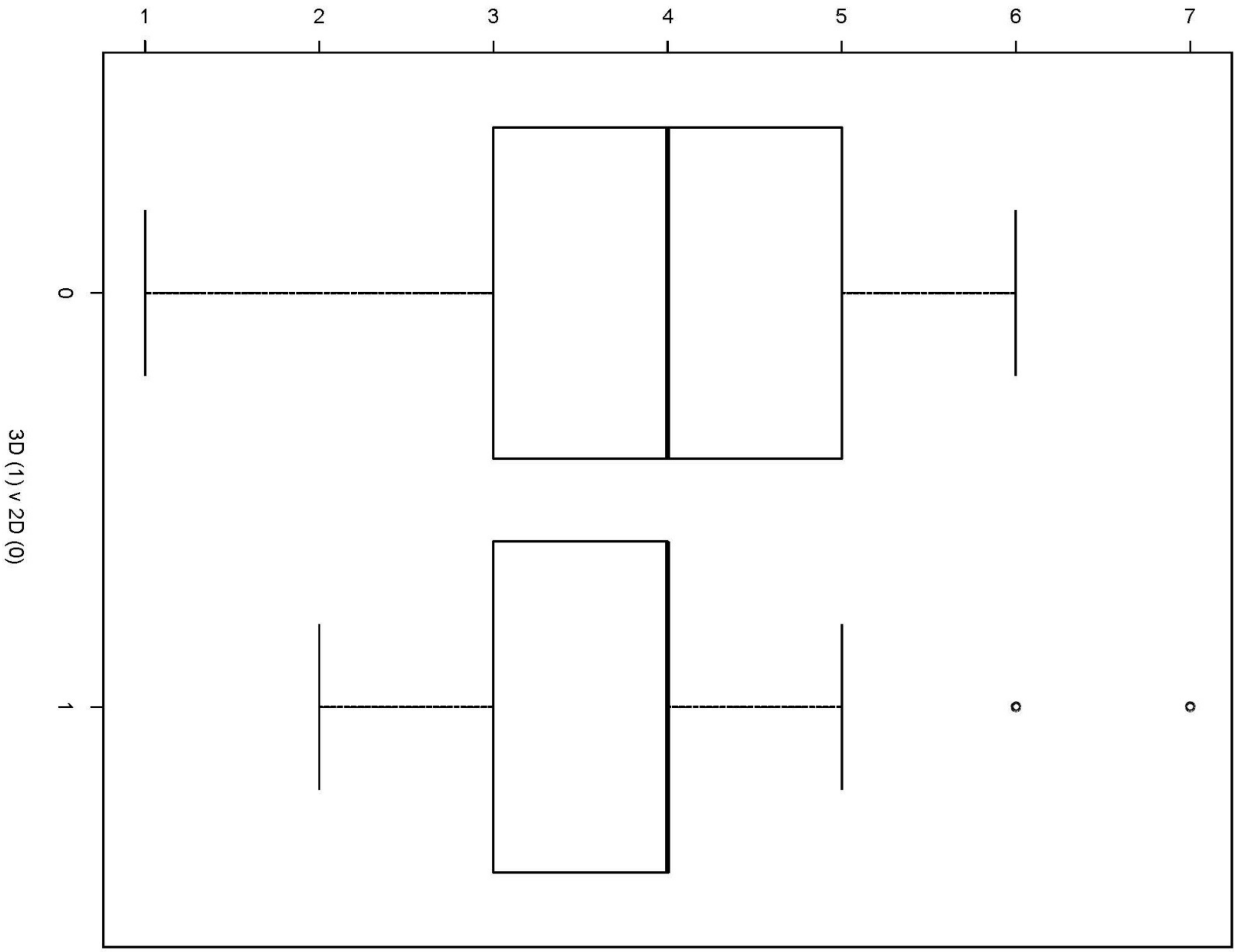

© 2020 Gabrielli ME et al. *JAMA Network Open.*
